# A decomposition analysis of change in skilled birth attendants, 2003 to 2008, Ghana demographic and health surveys

**DOI:** 10.1186/s12884-014-0415-x

**Published:** 2014-12-24

**Authors:** Samuel Bosomprah, Genevieve Cecelia Aryeetey, Justice Nonvignon, Richard M Adanu

**Affiliations:** Department of Biostatistics, School of Public Health, University of Ghana, Legon, Accra, Ghana; Department of Health Policy, Planning and Management, School of Public Health, University of Ghana, Legon, Accra, Ghana; School of Public Health, University of Ghana, Legon, Accra, Ghana

## Abstract

**Background:**

The single most critical intervention to improve maternal and neonatal survival is to ensure that a competent health worker with midwifery skills is present at every birth, and transport is available to a referral facility for obstetric care in case of an emergency. This study aims to describe changes in percentage of skilled birth attendants in Ghana and to identify causes of the observed changes as well as the contribution of different categories of mother’s characteristics to these changes.

**Method:**

This study uses two successive nationally representative household surveys: the 2003 and 2008 Ghana Demographic and Health Surveys (GDHS). The two datasets have comparable information on household characteristics and skilled attendants at birth at the time of the survey. The 2003 GDHS database includes information on 6,251 households and 3639 live births in the five years preceding the survey, whereas the 2008 GDHS database had information on11, 778 households and 2909 live births in the five years preceding the survey. A decomposition approach was used to explain the observed change in percentage of skilled birth attendants. Random-effects generalized least square regression was used to explore the effect of changes in population structure in respect of the mother’s characteristics on percentage of skilled birth attendants over the period.

**Results:**

Overall, the data showed absolute gain in the proportion of births attended by a health professional from 47.1% in 2003 to 58.7% in 2008, which represents 21.9% of gap closed to reach universal coverage. The increase in skilled birth attendants was found to be caused by changes in general health behaviour. The gain is regardless of the mother’s characteristics. The structural change in the proportion of births in respect of birth order and mother’s education had little effect on the change in percentage of skilled birth attendants.

**Conclusion:**

Improvement in general health behaviour can potentially contribute to an accelerated increase in proportion of births attended by skilled personnel in Ghana.

**Electronic supplementary material:**

The online version of this article (doi:10.1186/s12884-014-0415-x) contains supplementary material, which is available to authorized users.

## Background

Improvement in maternal and neonatal survival remains a global priority. Globally, the total number of maternal deaths decreased from 543,000 in 1990 to 287,000 in 2010 [1]. Likewise, global maternal mortality ratio declined from 400 maternal deaths per 100,000 live births in 1990 to 210 in 2010. This represents an average annual decline of 3.1% [1]. In Ghana, maternal survival has improved over the past 20 years albeit at a slow pace. According to the Maternal Mortality Estimation Inter-Agency Group, maternal mortality ratio reduced from 580 per 100,000 live births in 1990 to 350 per 100,000 live births in 2010, representing a 40% reduction [1]. Three-quarters of all maternal deaths occur during delivery and the immediate post-partum period [2]. The common direct causes of maternal death in sub-Saharan Africa includes hemorrhage, sepsis, and pregnancy-induced hypertension [2]. On the other hand, neonatal mortality especially early neonatal mortality covering deaths in the first seven days after birth is of interest because the health interventions needed to address it generally relate to the mother. The major causes of early neonatal deaths in developing countries include prematurity, birth asphyxia, and infections [3].

The single most critical intervention to improve maternal and neonatal survival is to ensure that a competent health worker with midwifery skills is present at every birth, and transport is available to a referral facility for obstetric care in case of an emergency [4]. Increasing the proportion of births assisted by skilled personnel (Doctor, Nurse, Midwife, Auxiliary midwife, or Community health officer) is a central strategy for improving maternal and newborn survival in Ghana [4]. Skilled attendant at delivery is used as an indicator to track progress toward the Millennium Development Goal target of reducing maternal mortality by three-quarters between 1990 and 2015 [5]. According to the Ghana Demographic and Health Survey (GDHS), the percentage of skilled delivery increased from about 47% in 2003 to 59% in 2008 [6,7]. Studies have shown that household wealth is associated with the rate of skilled birth attendant [8,9]. Other household factors related to poverty have been shown as important barriers to skilled birth attendant - illiteracy is a well-known barrier to recognizing birth-related complications and seeking appropriate health care [10,11]. However, little is known about the sources of this increase in the percentage of skilled birth attendants. This study aims to fill this knowledge gap by identifying causes of the observed changes as well as the contribution of different categories of mother’s characteristics to these changes.

## Methods

### Data sources

This study used two successive nationally representative household surveys: the 2003 and 2008 GDHS [6,7]. The two datasets have comparable information on household characteristics and skilled attendants at birth at the time of the survey. The survey was designed to provide information to monitor the population and health situation in Ghana. The survey used a two-stage sample design to produce separate estimates for key indicators for each of the ten regions in Ghana. The first stage involved selecting clusters (called enumeration areas) from an updated master sampling frame constructed from the recent Ghana Population and Housing Census [12]. A complete household listing operation was conducted in all the selected clusters to provide a sampling frame for the second stage selection of households. The second stage of selection involved the systematic sampling of the households listed in each cluster. Each household selected was eligible for interview. In these surveys a household was defined as a person or a group of persons, related or unrelated, who live together in the same house or compound, share the same housekeeping arrangements, and eat together as a unit. Further details of the sample design and questionnaire are described elsewhere [6,7].

The 2003 GDHS database included information on 6,251 households and 3639 live births in the five years preceding the survey, whereas the 2008 GDHS database had information on 11, 778 households and 2909 live births in the five years preceding the survey. The two surveys offered the opportunity for analysing coverage trends in the proportion of births attended by a skilled professional.

### Variables

The exposure of interest for this study include mother’s age at birth, birth order, maternal education [10,11], and socioeconomic status measured as household wealth index [8,9]. The household wealth index was estimated using an asset index. The asset index was constructed based on housing characteristics, household assets and possession of household consumer durables as well as access to clean water and improved sanitation using Principal Component Analysis technique, developed by Filmer and Pritchett [13]. Using rank methods, households were classified by wealth quintiles. The percentage of skilled birth attendants was defined as the proportion of live births in the five years preceding the survey delivered with the assistance of a skilled health professional (i.e. Doctor, Nurse, Midwife, Auxiliary midwife, or Community health officer).

### Statistical analysis

Examining coverage trends is essential for assessing country progress. Information on trends requires at least two separate and comparable measurements at two points in time. A measure of progress - coverage gap - defined as how much coverage would need to increase from 2003 level to reach universal coverage was estimated to examine coverage trends. The change from 2003 to 2008 was then expressed as a percentage of this gap.

To explain the observed change in percentage of skilled birth attendants, the decomposition approach was used. Several regression decomposition approaches exist in the literature. The conventional Blinder-Oaxaca [14,15] decomposition is based on two linear regression models that are fitted separately for the groups A and B:

$$ {Y}_A={\boldsymbol{X}}_A{\boldsymbol{b}}_A+{e}_A $$

$$ {Y}_B={\boldsymbol{X}}_B{\boldsymbol{b}}_B+{e}_B $$

For these models, Blinder [14] and Oaxaca [15] proposed the decomposition equations:

$$ {Y}_A-{Y}_B=\left({\boldsymbol{X}}_A-{\boldsymbol{X}}_B\right){\boldsymbol{b}}_A+{\boldsymbol{X}}_B\left({\boldsymbol{b}}_A-{\boldsymbol{b}}_B\right) $$

and

$$ {Y}_A-{Y}_B=\left({\boldsymbol{X}}_A-{\boldsymbol{X}}_B\right){\boldsymbol{b}}_B+{\boldsymbol{X}}_A\left({\boldsymbol{b}}_A-{\boldsymbol{b}}_B\right) $$

where *Y*_*A*_ − *Y*_*B*_ is the mean outcome difference, and ***X***_*A*_ and ***X***_*B*_ are mean vectors of the estimated coefficient vectors ***b***_*A*_ and ***b***_*B*_ for the two groups. In both equations, the first term on the right-hand side displays the difference in the outcome variable between the two groups due to differences in observable characteristics, whereas the second term shows the differential that is due to differences in coefficient estimates.

The approach used in this paper divides the change in percentage of skilled birth attendants into change in population structure and change in health behaviour and/or public health over the two time periods (or groups) 2003 to 2008 [16,17]. The population structure was defined as the ratio of number of births in each category or level of the exposure of interest to the sample size expressed as a percentage. The decomposition analyses were performed using national level data disaggregated by birth order, maternal education, and household wealth index. This method assumes that the historical change in the proportion of births attended by skilled professional depends on: 1) change in distribution of maternal education, birth order, maternal age and household wealth index over time (i.e. composition effect); 2) actual change in the proportion of births attended by skilled professional due to change in health behavior or improvement in public health (i.e. basic effect - the regression intercept when x = 0 (**α**)); and 3) variation of the proportion of births attended by skilled professional by exposure variables (**β**), and the residual effect of other variables not considered as the error term (μ) [16]. This can be specified mathematically as follows:

$$ \varDelta S=\left[{\displaystyle \sum }{\overline{s}}_j*\varDelta {w}_j\right]+\left[{\displaystyle \sum }{\overline{w}}_j*\varDelta \alpha \right]+\left[{\displaystyle \sum }{\overline{w}}_j*x\varDelta \beta \right]+\left[{\displaystyle \sum }{\overline{w}}_j*x\varDelta \mu \right] $$

where Δ denotes change,

*S* = percentage of skilled birth attendants,

${\overline{s}}_j$ = arithmetic mean of percentage of skilled birth attendants for the *j*^*th*^ category of the exposure variable,

*w*_*j*_ = the population structure for the *j*^*th*^ category of the exposure variable.

${\overline{w}}_j$ = arithmetic mean of the population structure for the *j*^*th*^ category of the exposure variable,

*Δw*_*j*_ = change in population structure expressed as a fraction for the *j*^*th*^ category of the exposure variable, and,

*x* = the level of an exposure variable, e.g. maternal age was categorized as 1 (<20 years), 2 (20–34 years), and 3 (35–49 years). So *x =* 1, 2, 3.

The parameters in the mathematical model were estimated using an Excel spreadsheet program developed for this analysis and it is attached as an Additional file 1 to this manuscript [16].

In a separate analysis, the effect of changes in population structure in respect of the mother’s characteristics (i.e. exposure of interest) on percentage of skilled birth attendants over the period was explored using the random-effects generalized least squares (GLS) regression. Independent panel datasets were constructed for each of the characteristics (birth order, mothers education, and household wealth index). The panel was defined as the category of the exposure of interest and the order of observations within panel was considered ordered by the number of surveys (two in this case). For example, the birth order and maternal education datasets each contains eight observations, while the household wealth index dataset contains 10 observations. Each dataset contains the following variables: the panel identifier, time identifier, proportion of births in each category of the characteristics (i.e. population structure), and percentage of skilled birth attendants. The analyses were performed with Stata 12 for Mac (StataCorp, College Station, USA) [18].

The random-effects GLS regression model can be specified as follows:

$$ {y}_{it}=\alpha +\beta {w}_{it}+{v}_i+{\epsilon}_{it} $$

In this model, *v*_*i*_ + *ϵ*_*it*_ is the residual that we have little interest in; the interest is to estimate *β* (the effect of change in the structure of the exposure of interest). *w*_*it*_ is the population structure of the exposure of interest in the *i*^th^ panel at survey time, *t. v*_*i*_ is the unit-specific residual; it differs between units, but for any particular unit, its value is constant. *ϵ*_*it*_ is the “usual” residual with the usual properties (mean 0, uncorrelated with itself, uncorrelated with *w*, uncorrelated with *v* , and homoskedastic) [18].

In all analyses, key survey characteristics such as sampling weight, stratification and clustering were accounted for.

### Ethics statement

Ethical approval for the surveys was obtained from the Ghana Health Service. All participants gave a verbal or written consent by either appending their signature or thumb printed the consent form. Parental consent was obtained for children and where the child was capable, an assent was obtained from the child in addition to parental consent. Participants were informed about the aim of the study. Personal identifiers were not taken as part of data collection. The data available for this study cannot be linked to an individual who participated in the study. Approval was granted by Macro International to use the data for this study.

## Results

### Sample description and changes in percentage of skilled birth attendants

The 2008 GDHS recorded 2,909 births compared with 3,639 births in 2003. The more educated a woman was, the more likely she was to have delivered with the assistance of a skilled attendant but this population structure has changed over the period (Table 1). For instance, the proportion of births among mothers with no education decreased from 40.3% (1,466/3639) in 2003 to 32.7% (952/2909) in 2008 and that among mothers with secondary or higher education increased from 5.2% (191/3639) in 2003 to 9.1% (263/2909) in 2008. Delivery assistance by a health professional showed little association with women’s age but it was related to the number of children a woman had. The more children a woman had the less likely she was to have a health professional attending her delivery. This structure had changed slightly over the period. For example, in 2003 about 20% (726/3639) of women had six or more children compared with 15.6% (455/2909) in 2008. Overall, the data showed absolute gain in the proportion of births attended by a health professional from 47.1% in 2003 to 58.7% in 2008. This gain represents 21.9% of gap closed to reach universal coverage (Table 1).

**Table 1:** **Changes in percentage of skilled birth attendants in the five years preceding the survey, 1998–2008, Ghana**

**Characteristics**	**GDHS2003 (1998–2003)**		**GDHS2008 (2003–2008)**		**Proportion of gap closed** ^**2**^
	**Number of birth (% of Total)**	**Percentage of skilled birth attendants** ^**1**^ **(A)**	**Number of birth (% of Total)**	**Percentage of skilled birth attendants** ^**1**^ **(B)**	
**Maternal age**					
<20	411 (11.3)	48.4	333 (11.4)	52.2	7.4
20-34	2,507 (68.9)	47.7	2,079 (71.5)	60.6	24.7
35-49	720 (19.8)	44.0	497 (17.1)	54.9	19.5
**Birth order**					
1	820 (22.5)	59.9	688 (23.7)	70.7	26.9
2-3	1,271 (34.9)	48.2	1,107 (38.1)	60.6	23.9
4-5	822 (22.6)	41.6	659 (22.7)	53.8	20.9
6+	726 (20.0)	36.8	455 (15.6)	42.6	9.2
**Mother’s education**					
None	1,466 (40.3)	29.7	952 (32.7)	36.3	9.4
Primary	843 (23.2)	44.4	722 (24.8)	54.6	18.3
Middle/JHS*	1,139 (31.3)	64.3	970 (33.4)	74.4	28.3
Secondary+	191 (5.2)	89.4	263 (9.1)	92.4	28.3
**Wealth Index**					
Poorest	941 (25.9)	20.6	744 (25.6)	24.2	4.5
Second	809 (22.2)	31.9	641 (22.0)	50.0	26.6
Middle	721 (19.8)	43.3	549 (18.9)	64.8	37.9
Fourth	617 (17.0)	73.1	560 (19.3)	81.7	32.0
Richest	551 (15.1)	90.4	415 (14.3)	94.6	43.8
**Total**	3639 (100)	47.1	2909 (100)	58.7	21.9

^1^Skilled provider includes doctor, nurse, midwife, auxiliary midwife, and community health officer.

^2^% gap closed = [(B)-(A)]*100/[100-(A)].

Note: % of Total represents the population structure.

*JHS represents junior high school.

### Decomposition of change in percentage of skilled birth attendants

The change in percentage of skilled birth attendants was decomposed by birth order, mother’s education and household wealth index. This decomposition may contribute to the understanding of how the observed changes related to variations in the survey population structure or to changes in public health and/or changes in behaviour. In general, the decomposition results indicated that health behaviour were the principal source of the gain in percentage of skilled birth attendants between 2003 and 2008 regardless of the exposure variable (Table 2, column Total). The analysis of behavioral effect suggested that the observed gain in percentage of skilled birth attendants was global (not specific to some socioeconomic characteristics). In other words, the observed changes were due to the general improvement in health behaviour in Ghana. The differentiation effect, the error terms, and composition effect were negligible. The contributions of each socioeconomic category in the overall gain in percentage of skilled birth attendants were also presented (Table 2, column Contribution). Depending on the exposure variable, the increase in percentage of skilled birth attendants in the following groups had contributed more to the observed changes: babies whose mothers had Middle or Junior High School education (40.7%), babies whose mothers had two to three births (54%), and babies born to “middle” class families (Table 2, column Contribution). It is however interesting to note that babies born to “richest” class families (Contribution = −1.1%) and those whose mothers had six or more births (Contribution = −6.2%) did not contribute to the observed change.

**Table 2:** **Decomposition of changes in percentage of skilled birth attendants, 1998–2008, Ghana**

	**Composition**	**Behaviour effect**				
		**Base**	**Differentiation**	**Error**	**Total**	**Contribution**
**Birth order**						
1	0.784	2.911	−0.347	−0.069	2.495	28.3%
2-3	1.741	4.599	−1.095	1.022	4.526	54.0%
4-5	0.048	2.854	−1.019	0.929	2.763	24.2%
6+	−1.747	2.243	−1.068	−0.142	1.032	−6.2%
Overall	7.1%	108.3%	−30.3%	14.9%	92.9%	100%
**Mother’s education**						
None	−2.508	3.322	−0.401	−0.511	2.409	−0.9%
Primary	0.792	2.184	−0.528	0.792	2.448	27.9%
Middle/JHS*	1.456	2.944	−1.068	1.391	3.267	40.7%
Secondary+	3.545	0.651	−0.315	−0.122	0.215	32.4%
Overall	28.3%	78.3%	−19.9%	13.3%	71.7%	100%
**Household wealth index**						
Poorest	−0.067	3.311	−0.206	−2.178	0.927	7.4%
Second	−0.082	2.842	−0.354	1.512	4.000	33.8%
Middle	−0.486	2.488	−0.464	2.136	4.160	31.7%
Fourth	1.780	2.334	−0.581	−0.192	1.561	28.8%
Richest	−0.740	1.890	−0.588	−0.685	0.617	−1.1%
Overall	2.9%	110.2%	−18.8%	5.1%	96.5%	100%

*JHS represents junior high school.

### Effect of change in population structure on percentage of skilled birth attendants

The structural change in the proportion of births in respect of birth order and mother’s education had little effect on the change in percentage of skilled birth attendants. Specifically, there was no evidence of the effect of structural change in the proportion of births in respect of birth order (coefficient = 0.58; 95% CI = −0.88, 2.04; P = 0.44) and mother’s education (coefficient = −0.96; 95% CI = −2.26, 0.33; P-value = 0.14) on change in percentage of skilled birth attendants (Table 3). There was however strong evidence that structural change in the proportion of births in respect of socioeconomic status measured by household wealth index had significant effect on the change in percentage of skilled birth attendants (coefficient = −6.45; 95% CI = −8.10, −4.79; P-value <0.0001).

**Table 3:** **Random-effect generalised least square regression of the effect of change in population structure on percentage of skilled birth attendants**

**Change in proportion of births in respect of:**	**Change in percentage of skilled birth attendants (95%CI)**	**Z statistic; P-value**	***v***	***ε***
Birth order	0.58 (−0.88, 2.04)	0.78; 0.44	10.68	8.44
Mother’s education	−0.96 (−2.26, 0.33)	−1.5; 0.14	17.68	6.57
Household wealth index	−6.45 (−8.10, −4.79)	−7.6; <0.0001	0	10.52

## Discussion

This study aims to describe changes in percentage of skilled birth attendants and to identify causes of the observed changes as well as the contribution of different categories of mother’s characteristics to these changes. The results showed that the gain in percentage of skilled birth attendants observed in Ghana between 2003 and 2008 is regardless of the mother’s characteristics, suggesting that the observed changes are due to the general improvement in health behaviour. The findings are consistent with studies, which showed that socio-cultural factors are important predictors of skilled delivery. For example, in a literature review of the determinants of delivery service use, Gabrysch and Campbell 2009 found that perceived benefit and need affect the decision to seek delivery care [19]. The health behaviour especially the perceived benefit and need relates to a number of factors influencing the perception of how a facility delivery with skilled attendants would benefit mother and newborn and or how big the personal need for such care is. This perception is shaped by general awareness of the dangers of childbirth and interventions available at health facilities. It is also shaped by individual past experiences with pregnancy, childbirth and health services, as well as by risk assessment of the index pregnancy.

Specific knowledge about the risks of childbirth and the benefits of skilled attendant at delivery can increase preventive care seeking. Similarly, recognition of danger signs and knowledge about availability of key maternal and newborn interventions can increase care seeking for obstetric complications. Most studies like the GDHS on use of delivery care are cross-sectional and it is difficult to establish temporal effect. This then is a limitation of such studies. Studies have shown that women who know danger signs in pregnancy or who are told about complications at antenatal care are more likely to deliver in a health facility [20,21]. The 2008 GDHS collected qualitative data on socio-cultural factors impeding women to seek skilled care when pregnant and the results showed that the major concerns included getting money for treatment, availability of drugs as well as the availability of a health care provider [6]. Other factors mentioned were in respect of having to seek permission to go for treatment as well as lack of a female provider and not wanting to go alone. These findings corroborate the important contribution of general health behaviour in the observed change in percentage of skilled birth attendants in Ghana over the period 2003 to 2008.

The results have implications for consideration of culturally acceptable options that increase demand or use of health workers with lesser skill levels. The creative alternatives such as the community-based health planning and services or use of mobile teams of at least one female community health officer that can provide services should be consolidated. Communication for behavioral Impact [22] strategies would facilitate new ways of providing maternity services by increasing awareness and demand for safe care during delivery. Antenatal care (ANC) visits constitute one of the few times women in many resource-poor settings seek care for their own health [23], and, represent an important opportunity to help women best prepare for birth, as well as inform them about pregnancy-related complications, and the advantages of skilled delivery care [24,25]. Several studies showed that women who attend ANC are more likely to seek skilled delivery care [26-29].

## Conclusion

Improvement in general health behavior can potentially contribute to an accelerated increase in proportion of births attended by skilled personnel in Ghana. Skilled delivery care would translate into improved maternal and newborn survival.

### Availability of supporting data

The data used for this study can be accessed through the following links:

http://www.measuredhs.com/publications/publication-FR221-DHS-Final-Reports.cfm.

http://www.measuredhs.com/publications/publication-FR152-DHS-Final-Reports.cfm.

## Additional file

Additional file 1:
**Decomposition analysis spreadsheet 21 Sept 2014.**
